# Retrospective Analysis on the Gallstone Disease after Gastrectomy for Gastric Cancer

**DOI:** 10.1155/2015/827864

**Published:** 2015-06-09

**Authors:** Kyong-Hwa Jun, Ji-Hyun Kim, Jin-Jo Kim, Hyung-Min Chin, Seung-Man Park

**Affiliations:** ^1^Department of Surgery, St. Vincent's Hospital, College of Medicine, The Catholic University of Korea, Seoul 442-723, Republic of Korea; ^2^Department of Surgery, Incheon St. Mary's Hospital, College of Medicine, The Catholic University of Korea, Seoul 403-720, Republic of Korea

## Abstract

*Background*. The aim of this study is to evaluate the incidence of gallstone after gastrectomy, risk factors for gallstone formation, and the surgical outcome of cholecystectomy after gastrectomy. *Methods*. A total of 2480 gastric cancer patients who underwent curative resection at two institutions between January 1997 and December 2012 were retrospectively reviewed. The patients' age, gender, diabetes mellitus, type of gastrectomy, extent of node dissection, and type of reconstruction were evaluated. *Results*. Gallstone formation occurred in 128 of 2480 (5.2%) patients who had undergone gastrectomy for gastric cancer. The incidence of gallstones was significantly higher after total compared with subtotal gastrectomy. Roux-en-Y reconstruction and lymph node dissection in the hepatoduodenal ligament were associated with a significantly higher incidence. In multivariate analysis, diabetes mellitus and reconstruction method were identified as significant risk factors for gallstone development. The proportion of silent stone was higher in the laparoscopic cholecystectomy (LC) group than in the open cholecystectomy (OC) group. Operation time and hospital stay were shorter in the LC group than in the OC group. *Conclusions*. Diabetes mellitus and Roux-en-Y reconstruction are risk factors for gallstones after gastrectomy. Only a few postoperative complications after subsequent cholecystectomy occurred, even when using a laparoscopic approach.

## 1. Introduction

The incidence of gallstones is known to be high in patients who have undergone gastrectomy [1–5]. The underlying pathophysiology of this phenomenon includes resection of the anterior hepatic branch of the vagal nerve, nonphysiological reconstruction of the gastrointestinal tract, and altered response to and secretion of cholecystokinin [4, 6, 7]. Subsequent cholecystectomy after gastrectomy is challenging because of adhesion around Calot's triangle, chronic nutritional insufficiency, and intestinal reconstruction that interferes with access to the common bile duct (CBD). The higher incidence of gallstones and surgical obstacles have led to a randomized multicenter controlled trial, known as the Cholegas study, in which prophylactic cholecystectomy during gastrectomy for cancer was not found to increase the risk of perioperative morbidity, mortality, or cost [8]. However, not all patients with gallstones develop gallstone-related symptoms or require additional surgical treatments, and subsequent cholecystectomy using either an open or laparoscopic technique is feasible even in those patients who have undergone previous gastrectomy [1, 2, 9, 10]. Moreover, the current consensus recommends that only patients with symptomatic gallstones should undergo cholecystectomy, because asymptomatic patients rarely develop serious biliary complications [11, 12]. There have been several studies evaluating cholecystectomy in patients with a history of gastrectomy [1, 3, 5, 9]; however, few studies with sufficient data are available about risk factors for gallstone after gastrectomy and the surgical outcomes following cholecystectomy for gallstone disease.

In this study, we analyzed the incidence of gallstone formation after gastrectomy, the interval between gastrectomy and diagnosis of a gallstone, and risk factors for gallstone development. We also explored the surgical outcomes following cholecystectomy according to the surgical approach.

## 2. Methods

### 2.1. Patients

**Figure 1:**
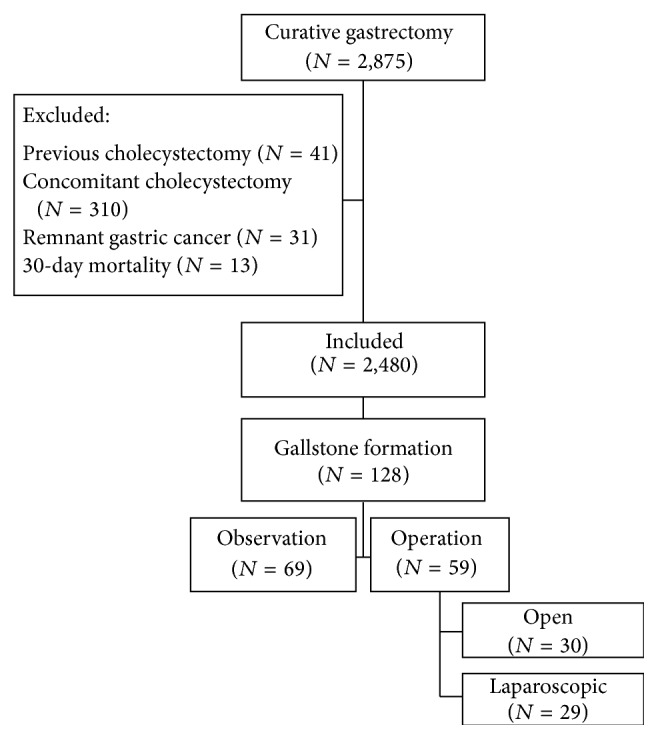
Patient enrollment and outcomes.

We enrolled 2875 patients who underwent curative gastric cancer surgery at the Department of Surgery, Incheon St. Mary's Hospital and St. Vincent's Hospital, The Catholic University of Korea between January 1997 and December 2012. Exclusion criteria were (1) previous cholecystectomy (*n* = 41), (2) prophylactic cholecystectomy (*n* = 310), (3) remnant gastric cancer (*n* = 31), and (4) mortality within 30 days after surgery (*n* = 13) (Figure 1). After applying these criteria, a total of 2480 patients were included in this study. All 2480 cases involved either total gastrectomy or distal gastrectomy associated with systemic lymph node dissection. Open gastrectomy was performed in 1488 cases and laparoscopic gastrectomy was performed in 992 cases. Lymph node dissection and TNM staging were performed following the guidelines of the Japanese Research Society for Gastric Cancer (JRSGC) [13]. Every patient underwent vagotomy associated with gastrectomy and lymph node dissection. Intestinal reconstruction was performed using Roux-en-Y method for total gastrectomy and Billroth I or II anastomosis or Roux-en-Y reconstruction for distal gastrectomy. The patients were followed for a median of 91.6 (range, 15–206) months. Data from these patients were entered into a prospectively maintained database. The Institutional Review Boards of Incheon St. Mary's Hospital (OC14RIMI0034) and St. Vincent's Hospital (VC14RIMI0046) approved the study protocol.

The patients were followed up with an abdominal computed tomography (CT) scan every 6 months for 2 years after gastrectomy, every year for up to 5 years, and then every year until the end of the study period or death. The primary objective of such examinations was the detection of metastatic disease, but gallbladder information was recorded in every examination. Ultrasound examination was also performed if gallstone disease was detected by abdominal CT or if biliary pain occurred. Indications for surgery were biliary pain and biliary complications, such as acute cholecystitis, choledocholithiasis, cholangitis, or pancreatitis. Cholecystectomy was performed whenever the indications for surgery were appropriate or when patients with silent gallstone disease requested surgery using either an open or laparoscopic surgical approach.

The patients were classified into two subgroups: stone negative (SN, *n* = 2352) and stone positive (SP, *n* = 128). The incidence of gallstone formation after gastrectomy for gastric cancer and the influence of clinical and surgical factors on stone formation were analyzed. The open cholecystectomy (OC) and laparoscopic cholecystectomy (LC) groups were also retrospectively compared with respect to the interval between gastrectomy and detection of a gallstone, the duration between detection of gallstones and cholecystectomy, indication of surgery, operation time, blood loss, postoperative complications, and hospital stay.

### 2.2. Statistics

Continuous variables were presented as means and standard deviations, while categorical variables were presented as frequencies and percentages. Fisher's exact test was used to compare differences in discrete or categorical variables, and the Mann-Whitney test was used for continuous variables. The cumulative incidence of gallstones after gastrectomy was evaluated by the Kaplan-Meier method and differences between groups were evaluated by the log rank test. Multivariate analysis with Cox proportional hazards model was used to compare the influence of each type of lymph node dissection and the type of reconstruction performed. Differences were considered statistically significant when the *P* value was <0.05. Statistical analysis was done using SPSS version 20 (SPSS, Chicago, IL, USA).

## 3. Results

### 3.1. Demographic Data of Gastric Cancer Patients Who Underwent Curative Resection

**Figure 2:**
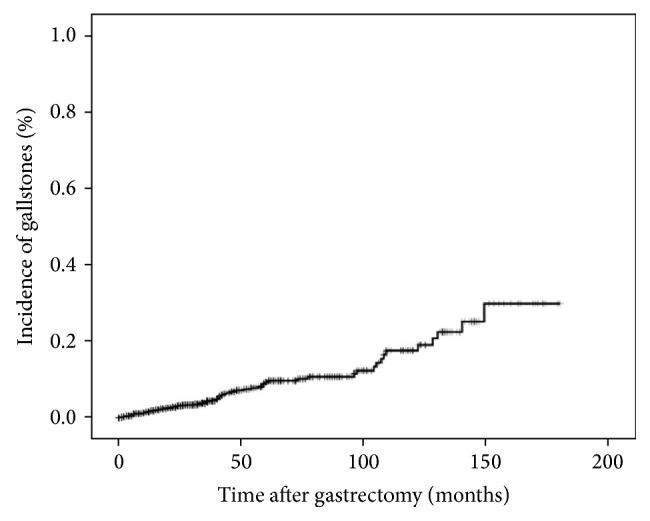
Incidence of gallstones after gastrectomy.

The characteristics of 2480 patients are summarized in Table 1. One hundred twenty-eight patients were diagnosed with gallstones. The incidence of gallstones at 5 and 10 years after gastrectomy was 11.4 and 19.1 percent, respectively (Figure 2). The interval between gastrectomy and diagnosis of gallstones was 45.9 months (range 1–149 months). Ninety-three of the 128 cases (72.7%) were diagnosed within 5 years after gastrectomy. The age of the 2352 patients (1554 men and 798 women) in the stone negative (SN) group was 59.9 ± 11.7 years and that of the 128 patients (80 men and 48 women) in the stone positive (SP) group was 60.6 ± 12.9 years. There were no significant differences in age (*P* = 0.539), gender (*P* = 0.407), BMI (*P* = 0.663), or diabetes mellitus (*P* = 0.095) between these two groups. With respect to types of gastrectomy, the proportion of total gastrectomy was higher in SP group than in SN group. Furthermore, the proportion of Roux-en-Y reconstruction and lymph node dissection in the hepatoduodenal ligament were higher in SP group than in SN group. Depth of invasion, lymph node metastasis, and TNM stage did not differ significantly between the two groups.

### 3.2. Risk Factors for Gallstones after Gastrectomy

The incidence of gallstone formation is shown in Table 2. The incidence of gallstones was significantly higher after total gastrectomy than after subtotal gastrectomy. Those with Roux-en-Y reconstruction were more likely to develop gallstones after gastrectomy than those with Billroth I or II reconstruction. Dissection of lymph node 12 correlated significantly with the development of gallstones. There were no significant differences in age, sex, or diabetes mellitus between the two groups. Multivariate analysis using the Cox proportional hazard model was carried out. Roux-en-Y reconstruction was identified as a significant risk factor for gallstones after gastrectomy, along with diabetes mellitus.

### 3.3. Surgical Treatment for Gallstone Disease after Gastrectomy

Of the 128 patients with gallstones, 59 (46.1%) underwent surgical treatment for gallstone disease (Table 3). Open cholecystectomy (OC) was performed in 30 patients (50.8%) and laparoscopic cholecystectomy (LC) in 29 patients (49.2%). There was 1 open conversion case in the LC group due to dense adhesion and bleeding around Calot's triangle. Mean age in the LC group was younger than in the OC group (62.3 ± 2.4 versus 55.9 ± 2.2 years, resp.; *P* = 0.048). However, gender, body mass index, interval between gastrectomy and detection of stones, type of gastrectomy, and reconstruction after gastrectomy were not significantly different between the two groups. Indications of surgery were significantly different. The proportion of silent stone was higher in the LC group than in the OC group, whereas the proportion of common bile duct (CBD) stone or cholangitis is higher in the OC group than in the LC group. The interval between diagnosis of gallstones and cholecystectomy was not different between the two groups (11.7 ± 5.5 versus 11.8 ± 3.2 months, resp.; *P* = 0.936). Preoperative management of gallstones, such as percutaneous transhepatic biliary drainage (PTBD), percutaneous transhepatic gallbladder drainage (PTGBD), and endoscopic retrograde cholangiopancreatography (ERCP), was not significantly different between the two groups. CBD exploration was performed more frequently in the OC group than in the LC group. Operation time was shorter in the LC group than in the OC group. LC patients showed shorter hospital stay (*P* < 0.001) and lower incidence of complications (*P* = 0.003).

## 4. Discussion

To date, gallstone management decisions in patients who underwent gastrectomy are dependent only on the surgeon's preference and expertise. Prophylactic cholecystectomy has been proposed, because concomitant cholecystectomy is not time consuming, poses minimal risks for patients, and allows patients to avoid difficult procedures, such as ERCP, during intestinal reconstruction [9, 14–16]. Others have argued that patients with asymptomatic gallstones develop serious biliary symptoms at a low rate over time; thus, subsequent cholecystectomy after gastrectomy can be performed safely, and the additional calculated morbidity for subsequent cholecystectomy is lower than the morbidity for prophylactic cholecystectomy [1, 2, 9, 17]. In this study, 5.2% of patients undergoing gastrectomy were diagnosed with gallstones during follow-up, and only half of them required cholecystectomy. Moreover, there were no mortalities or major injuries as a result of a previous gastrectomy. Thus, concomitant cholecystectomy during gastrectomy is not necessary.

In this study, the incidence of gallstones was compared in subgroups of patients classified according to the type of gastrectomy. Gallstone formation was more frequent (10.1 versus 3.9%) in the total gastrectomy group than in the subtotal gastrectomy group. However, gallstones in the total gastrectomy group did not form earlier or require more frequent surgical treatment than those in the subtotal gastrectomy group. Destruction of vagal nerves is one of the risk factors for formation of gallstones after gastrectomy [4, 6, 7]. A previous experimental study showed that gastrectomy abolishes phasic contraction of gallbladder bile and mixing of gallbladder bile with fresh hepatic bile, leading to supersaturation and increased gallstone formation [18]. This suggests that complete amputation of the vagal trunk with dissection of the esophagus influences the contractile ability of the gallbladder.

Exclusion of the duodenum during reconstruction is known to be associated with the development of gallstones. Kobayashi et al. suggested that exclusion of the duodenum leads to changes in cholecystokinin secretion, resulting in decreased gallbladder contraction and an increased risk of gallstones [4]. They demonstrated that reconstruction with duodenal exclusion, such as Billroth II or Roux-en-Y, was associated with a significantly higher incidence than was nonexclusion. In this study, Roux-en-Y reconstruction was associated with a significantly higher incidence than Billroth I or II reconstruction. Kobayashi et al. also showed that patients who had lymph node dissection in the hepatoduodenal ligament had a significantly higher incidence of gallstones than those who did not [4]. In this study, patients who had lymph node dissection in the hepatoduodenal ligament had a significantly higher incidence of gallstones than those who did not. However, only Roux-en-Y reconstruction was identified as one of significant risk factors for gallstone development.

In this study, there were no severe biliary complications in patients who underwent subsequent cholecystectomy after gastrectomy. One patient in open cholecystectomy group experienced a postoperative bile leak but managed conservatively. In the Cholegas study, 12 patients in the prophylactic cholecystectomy (PC) group and six patients in the standard gastric surgery only (SS) group experienced surgical complications, and only one biliary complication was recorded in the PC group [8]. The study showed no statistical significance in surgical complications, even though surgical complications in the PC group were twice as common as those in the SS group. In contrast, Gillen et al. demonstrated that simultaneous cholecystectomy resulted in a higher morbidity of 0.95% compared with the calculated additional morbidity of early and late cholecystectomy of 0.45% [3]. They also suggested that removal of normal acalculous gallbladders during upper GI surgery should be recommended, because late cholecystectomy can be performed safely and has a lower morbidity than simultaneous cholecystectomy.

Laparoscopic cholecystectomy is a minimally invasive technique that has become the standard treatment for cholelithiasis, with many well-known advantages over open cholecystectomy [2, 19]. However, laparoscopic cholecystectomy is challenging after gastric surgery, because the extensive and dense adhesions around the gallbladder increase the risk of conversion, bile duct injuries, and a longer operation time [9, 15]. Kwon et al. reported that the conversion rate from laparoscopic to open surgery was 10%, and the overall complication rate was 14% in patients with a previous gastrectomy who underwent laparoscopic cholecystectomy [17]. Kim et al. analyzed cholecystectomy after gastrectomy and identified the factors related to surgical outcome of these associated procedures [20]. They found that the laparoscopic approach was not related to operation time but was related to a shorter hospital stay, while CBD exploration and laparoscopic surgery were not independently related to the occurrence of complications. In this study, LC group had shorter operation times, lower blood loss, and shorter hospital stays than OC group. These findings might result from more frequent rate of CBD exploration in the OC group than in the LC group.

There are some limitations of this study. First, there may have been bias in the patient selection, because our data were obtained retrospectively in two institutions. Regional or population distribution differences also cannot be disregarded. Second, we did not routinely perform follow-up ultrasound examination on a periodic basis; therefore, it is possible that the true number of patients with gallstones is higher as CT is recognized as being less accurate than ultrasound in detection of gallstones. Third, indications of surgery were significantly different between the two groups. High proportion of acute cholecystitis and CBD exploration in the OC group may increase the incidence of complications and operation time. Additional well-designed, large cohort studies are needed to clarify this issue.

In conclusion, the incidence of gallstone after gastrectomy was significantly higher in total gastrectomy, Roux-en-Y reconstruction, and lymph node dissection in the hepatoduodenal ligament. Diabetes mellitus and Roux-en-Y reconstruction were significant risk factors for gallstones after gastrectomy in multivariate analysis. Of 128 cases with gallstones that were followed for 91.6 ± 26.4 months, 81 patients (63.3%) remained asymptomatic. Moreover, only a few postoperative complications occurred after subsequent cholecystectomy, even when using a laparoscopic approach. Thus, prophylactic cholecystectomy might be unnecessary. However, to define more clearly the incidence, risk factors, and surgical outcomes of gallstone after gastrectomy, a controlled prospective clinical trial should be performed.

## Figures and Tables

**Table 1:** Characteristics of the 2480 patients.

	Stone negative after gastrectomy (*n* = 2,352)	Stone positive after gastrectomy (*n* = 128)	*P* value
Age	59.9 ± 11.7	60.6 ± 12.9	0.539
Gender			
Male	1554 (66.1)	80 (62.5)	0.407
Female	798 (39.9)	48 (37.5)	
BMI (Kg/m^2^)	24.8 ± 2.1	23.2 ± 3.5	0.663
DM			
No	2034 (86.5)	104 (81.3)	0.095
Yes	318 (13.5)	24 (18.8)	
Type of gastrectomy			
Total gastrectomy	436 (18.5)	49 (38.3)	<0.001
Subtotal gastrectomy	1916 (81.5)	79 (61.7)	
Reconstruction			
Billroth I	912 (38.8)	32 (25)	<0.001
Billroth II	745 (31.7)	36 (28.1)	
Roux-en-Y	695 (29.5)	60 (46.9)	
Extent of dissection			
D1+	752 (32)	36 (28.7)	0.418
D2	1600 (68)	92 (71.3)	
Number 12 LN dissection			
No	736 (31.3)	23 (17.9)	0.009
Yes	1616 (68.7)	105 (82.1)	
T stage			
pT1	1124 (47.8)	58 (45.3)	0.384
pT2	303 (12.9)	17 (13.3)	
pT3	254 (10.8)	20 (15.6)	
pT4	671 (28.5)	33 (25.8)	
LN metastasis			
Negative	1352 (57.5)	74 (57.8)	0.941
Positive	1000 (42.5)	54 (42.2)	
TNM stage			
I	1260 (53.6)	62 (48.4)	0.292
II	331 (14.1)	24 (18.8)	
III	761 (32.3)	42 (32.8)	

**Table 2:** Univariate and multivariate analysis of risk factors for gallstones after gastrectomy.

	Incidence of gallstones (%)	Univariate analysis	Multivariate analysis	
			Odds ratio	*P* value
Age (years)		0.105	1.021 (0.999–1.043)	0.060
Sex				
Male	4.9	0.346	1	
Female	5.7		1.275 (0.796–2.043)	0.313
DM				
No	4.9	0.110	1	
Yes	7.0		1.862 (1.033–3.357)	0.039
Type of gastrectomy				
Total gastrectomy	10.1	<0.001	1	
Subtotal gastrectomy	3.9		0.669 (0.324–3.275)	0.279
Reconstruction				
Billroth I	3.4	<0.001	1	
Billroth II	4.6		1.383 (0.698–2.740)	0.353
Roux-en-Y	7.9		3.936 (2.210–7.009)	<0.001
Number 12 LN dissection				
No	3.0	0.004	1	
Yes	6.1		0.806 (0.486–1.337)	0.404

**Table 3:** Surgical treatment for gallstone disease after gastrectomy.

	Open cholecystectomy (*n* = 30)	Laparoscopic cholecystectomy (*n* = 29)	*P* value
Age (year)	62.3 ± 2.4	55.9 ± 2.2	0.048
Gender			
Male	21 (70)	12 (44.8)	0.077
Female	9 (30)	16 (55.2)	
BMI (Kg/m^2^)	22.6 ± 3.9	23.8 ± 3.0	0.204
Interval between gastrectomy and detection of stones (month)	49.5 ± 6.9	50.5 ± 7.8	0.918
Type of gastrectomy			
Total gastrectomy	14 (46.7)	14 (48.3)	0.612
Subtotal gastrectomy	16 (53.3)	15 (51.7)	
Reconstruction			
Billroth I	4 (13.3)	5 (17.3)	0.417
Billroth II	11 (36.7)	7 (24.1)
Roux-en-Y	15 (50)	17 (58.6)
Indication of surgery			
Silent stone	1 (3.3)	11 (37.9)	0.001
Cholecystitis	12 (40)	14 (48.3)
CBD stone or cholangitis	17 (56.7)	4 (13.8)
Interval between detection of stones and cholecystectomy (month)	11.7 ± 5.5	11.8 ± 3.2	0.936
Operation			
Cholecystectomy	9 (30)	25 (86.2)	<0.001
Cholecystectomy and choledochotomy	21 (70)	4 (13.8)	
Preoperative management			
PTBD before cholecystectomy	1 (3.3)	1 (3.4)	0.981
PTGBD before cholecystectomy	2 (6.7)	0 (0)	0.157
ERCP before cholecystectomy	6 (20)	2 (6.9)	0.142
Operation time (min)	143 ± 8.8	82.7 ± 7.1	<0.001
EBL (mL)	218.5 ± 26.5	140.2 ± 30.7	0.059
Hospital stay (day)	10 ± 0.6	3.9 ± 0.4	<0.001
Complications	9 (30)	0 (0)	0.003
Postoperative bile leak	1 (3.3)	0 (0)
Wound infection	6 (20)	0 (0)
Pulmonary	2 (6.7)	0 (0)

PTBD = percutaneous transhepatic biliary drainage, PTGBD = percutaneous transhepatic gallbladder drainage, and ERCP = endoscopic retrograde cholangiopancreatography.
